# Ocular Involvement in Mantle Cell Lymphoma Detected by F 18 FDG PET/CT

**DOI:** 10.4274/MIRT.20.08

**Published:** 2011-04-01

**Authors:** Elgin Özkan, Seda Laçin, Çiğdem Soydal, Mine Araz, N. Özlem Küçük

**Affiliations:** 1 Ankara University, Faculty of Medicine, Department of Nuclear Medicine, Ankara, Turkey

**Keywords:** Lymphoma, non-Hodgkin; positron-emission tomography; eyelid disease

## Abstract

Mantle Cell Lymphoma (MCL) is an uncommon but aggressive form of non-Hodgkin’s lymphoma. The extranodal involvement is not unusual especially in bone marrow, spleen, gastrointestinal tract and Waldeyer’s ring. Ocular involvement is very exceptional and the most commonly affected site is the orbit (90%), followed by the lacrimal gland (50%) and the eyelids (50%). Today, PET/CT is widely used in non-Hodgkin’s lenfoma especially in staging and evaluation of treatment response. Authors report MCL with ocular involvement that was detected on PET/CT scan.

**Conflict of interest:**None declared.

## CASE REPORTS

**Figure 1:**
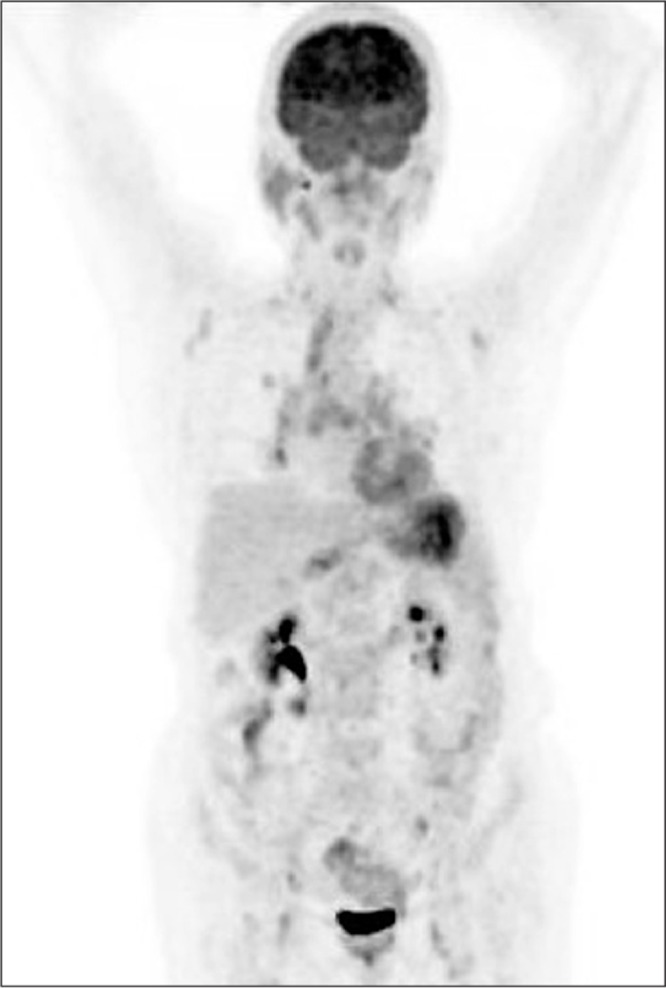
Maximum intensity projection (MIP) image of whole body in the third PET/CT scan. Pathological FDG uptake was seen at multiple lymph nodes in the head and neck, chest, abdomen and inguinal regions, and in the stomach

**Figure 2:**
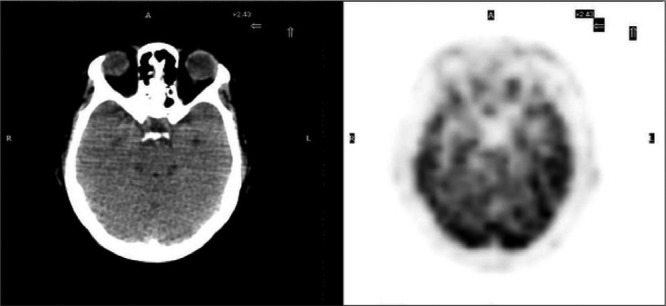
Axial CT and PET images of the orbita in the first PET/CT scan

**Figure 3:**
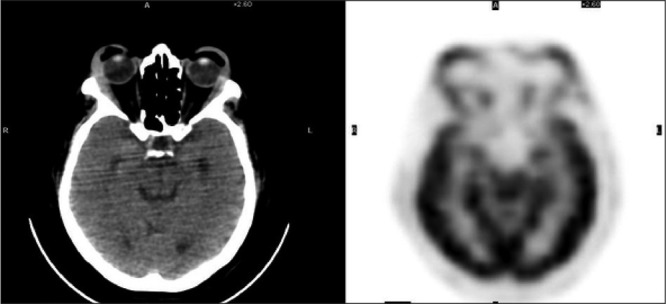
Axial CT and PET images of the orbita in the third PET/CT scan

Mantle Cell Lymphoma (MCL) has been diagnosed with scalene lymph node biopsy in a 63-year-old woman. At the time of diagnosis, the disease was at stage 4B with mediastinal, cervical, hilar, abdominal lymph nodes which were detected by CT (computerized tomography). There was also bone marrow involvement. She received chemotherapy and complete response was confirmed with control CT scans. The first PET/CT scan performed at this period was normal. She has been followed up disease free for about six months. Then she complained about cervical mass and bilateral eyelid swelling. Regarding to second PET/CT findings which was performed for restaging, she received chemotherapy again for the relapse in the cervical, mediastinal, hilar, abdominopelvic and inguinal lymph nodes. A slight FDG uptake was also seen at both eyelids on this second PET/CT. She was followed for a period of time without any medical treatment in accordance with the patient's request. Eight months later an inguinal lymph node was recognized and excisional biopsy revealed recurrence. She received of chemotherapy again and then third PET/CT was performed for restaging of the disease. In the maximum intensity projection (MIP) image of whole body, in the third PET/CT scan (Figure 1) showed pathological FDG uptake at multiple lymph nodes in the head and neck, chest, abdomen and inguinal regions, and diffusely in the stomach. Axial image of the orbita in the first PET/CT image showed no pathological ocular FDG uptake (Figure 2). After about 2 years, however, axial image of orbita in third PET/CT represented diffuse FDG uptake at both eyelids (Figure 3), significantly different from the first scan. There may be a physiological uptake at the extraocular muscles, but in this case, comparison of first and third PET/CT scan showed that the FDG uptake in the muscles can be easily recognized as pathological with increase in thickness of the muscles in CT (1).

MCL is an uncommon but aggressive form of non-Hodgkin’s lymphoma. The extranodal involvement is not unusual, especially in bone marrow, spleen, gastrointestinal tract and Waldeyer’s ring (2,3). Such localizations (breast, ocular adnexal region, skin etc.) are exceptional in MCL (4). The most commonly affected site of ocular involvement is the orbit (90%), followed by the lacrimal gland (50%) and the eyelids (50%) (5,6). MCL presenting in the ocular adnexal region has a male predominance and is associated with stage III/IV disease. Our stage IV patient did not have any ocular symptoms at diagnosis, however as the disease proceeded, severe eyelid edema occurred showing diffuse spread of the disease supported by PET/CT findings. We report an uncommon case with MCL; ocular involvement that was detected on PET/CT scan. This case reveals the need of total body PET/CT imaging in patients with aggressive non-Hodgkin’s lymphoma in order not to miss an exceptional involvement.
